# The therapeutic itinerary of health workers diagnosed with COVID-19*

**DOI:** 10.1590/1518-8345.4691.3413

**Published:** 2021-04-12

**Authors:** Liliane Faria da Silva, Emília Gallindo Cursino, Euzeli da Silva Brandão, Fernanda Garcia Bezerra Góes, Jéssica Renata Bastos Depiant, Laura Johanson da Silva, Rosane Cordeiro Burla de Aguiar

**Affiliations:** 1 Universidade Federal Fluminense, Escola de Enfermagem Aurora de Afonso Costa, Niterói, RJ, Brazil.; 2 Universidade Federal Fluminense, Departamento de Enfermagem, Rio das Ostas, RJ, Brazil.; 3 Universidade Federal do Rio de Janeiro, Escola de Enfermagem Anna Nery, Rio de Janeiro, RJ, Brazil.; 4 Scholarship holder at the Conselho Nacional de Desenvolvimento Científico e Tecnológico (CNPq), Brazil.; 5 Universidade Federal do Estado do Rio de Janeiro, Escola de Enfermagem Alfredo Pinto, Rio de Janeiro, RJ, Brazil

**Keywords:** Coronavirus Infections, Coronavirus, Pandemics, Health Services Accessibility, Universal Access to Health Care Services, Health Personnel, Infecções por Coronavírus, Coronavírus, Pandemias, Acesso aos Serviços de Saúde, Acesso Universal aos Serviços de Saúde, Pessoal de Saúde, Infecciones por Coronavirus, Coronavirus, Pandemias, Accesibilidad a los Servicios de Salud, Acceso Universal a los Servicios de Salud, Personal de Salud

## Abstract

**Objective:**

to analyze the therapeutic itinerary of health workers diagnosed with COVID-19.

**Method:**

qualitative study conducted with 132 health workers diagnosed with COVID-19. Data were collected using a semi-structured form sent through the social media and processed with the *Interface de R pour Analyses Multidimensionnelles de Textes et* de *Questionnaires*, according to the Descending Hierarchical Classification.

**Results:**

the participants included 116 women and 16 men with 14 different professions within the health field. Five classes of excerpts emerged from the text, revealing the therapeutic itinerary from the onset of symptoms, up to referrals for testing and confirming COVID-19. Additionally, the aspects that facilitated or hindered access to testing in healthcare units were identified, in addition to misinformation and the need for workers to pay for the tests to obtain a diagnosis.

**Conclusion:**

this study’s results show the difficulties health workers experienced to access the tests and related information and the delay in accessing the results and obtaining a sick leave to remain in isolation at home. The health workers who did not get support in terms of management and monitoring from the facilities where they worked adopted an active search.

## Abstract (pt)

**Objetivo:**

analisar o itinerário terapêutico de profissionais de saúde diagnosticados com COVID-19.

**Método:**

pesquisa qualitativa, desenvolvida com 132 profissionais de saúde diagnosticados com COVID-19. Dados foram coletados por meio de formulário semiestruturado, enviado via mídias sociais e processados no *software Interface de R pour Analyses Multidimensionnelles de Textes et de Questionnaires* pela Classificação Hierárquica Descendente.

**Resultados:**

dentre os participantes, havia 116 mulheres e 16 homens de 14 categorias diferentes da área da saúde. Obtiveram-se cinco classes de segmentos de texto que evidenciaram o itinerário terapêutico desde os primeiros sintomas, com apontamento dos encaminhamentos para a realização do teste de confirmação da COVID-19. Além disso, identificaram-se facilidades e dificuldades de acesso aos exames nas unidades de saúde, bem como desinformação e até pagamento dos testes para confirmação diagnóstica.

**Conclusão:**

os resultados do estudo denotam as dificuldades vivenciadas pelos profissionais de saúde no acesso aos testes e à informação sobre eles, além da demora dos resultados e do atendimento para os afastamentos funcionais, com vistas ao isolamento domiciliar. Evidenciou-se, ainda, a busca ativa por parte dos profissionais de saúde que estavam desamparados quanto ao necessário gerenciamento e monitoramento pelas instituições de trabalho.

## Abstract (es)

**Objetivo:**

analizar el itinerario terapéutico de profesionales sanitarios diagnosticados con COVID-19.

**Método:**

investigación cualitativa, desarrollada con 132 profesionales sanitarios diagnosticados con COVID-19. Se recopilaron datos a través de formulario semiestructurado enviado vía medios sociales y procesados en el *software Interface de R pour Analyses Multidimensionnelles de Textes et de Questionnaires* por Clasificación Jerárquica Descendente.

**Resultados:**

los participantes fueron 116 mujeres y 16 hombres de 14 categorías diferentes del área de la salud. Se encontraron cinco clases de segmentos de texto que evidenciaron el itinerario terapéutico desde los primeros síntomas, con indicación de los encaminamientos para la realización de la prueba de confirmación de la COVID-19. Además, se identificaron facilidades y dificultades de acceso a las pruebas en los servicios de salud, así como desinformación e incluso pago de las pruebas para confirmación diagnóstica.

**Conclusión:**

los resultados del estudio denotan las dificultades vividas por los profesionales de la salud en el acceso a las pruebas y a la información sobre ellas, además de la demora de los resultados y de la atención para alejamientos funcionales para fines del aislamiento domiciliario. Se evidenció, además, una búsqueda activa por parte de los profesionales de la salud que estaba desamparada en términos de la gestión y el monitoreo necesarios por las instituciones de trabajo.

## Introduction

In December 2019, the SARS-CoV-2 coronavirus, which causes the Coronavirus disease (COVID-19), was identified in the city of Wuhan (China) after a series of pneumonia cases. In February 2020, the virus had already spread to various countries, after which the World Health Organization (WHO) declared the pandemic^(^ 1 ^-^ 2 ^)^.

Currently, contamination by the SARS-CoV-2 is a very severe and challenging public health problem in many countries, including Brazil, with important consequences in the health, social, economic and political spheres. More than 7,600,000 cases and 427,000 deaths had been confirmed up to June 14^th^, 2020 worldwide and the numbers keep growing, with almost all countries reporting new cases daily^(^ 2 ^)^.

In Brazil, the first case was reported on February 26^th^, 2020 and the numbers are ascending ever since, reaching 828,810 confirmed cases and 41,828 deaths up to June 14^th^, 2020^(^ 2 ^)^. The first case was reported in Rio de Janeiro on March 5^th^, 2020 and, even though the state government imposed shelter in place measures on March 16^th^, 2020^(^ 3 ^)^, the number of individuals contaminated reached 79,572 on June 14^th^, 2020 and the state ranked second in Brazil in the number of cases^(^ 4 ^)^.

There is no complete information regarding the natural history of the virus or effective measures to clinical manage cases of human infection caused by the SARS-CoV-2 coronavirus. It is known, however, that the virus is highly transmissible and causes acute respiratory syndrome, ranging from mild to severe cases, with respiratory failure. Transmission mainly occurs through contact with respiratory droplets released by sick and symptomatic individuals^(^ 2 ^)^.

Transmission by asymptomatic individuals is still unclear^(^ 5 ^-^ 6 ^)^. On average, the period of incubation is 5-6 days, ranging from 0 to 14. The main signs and symptoms include fever, dry cough, dyspnea, myalgia or fatigue, upper respiratory symptoms and, more rarely, gastrointestinal symptoms^(^ 7 ^)^.

The diagnosis and isolation of infected individuals are important measures to prevent the dissemination of the virus and the contamination of new individuals. In terms of clinical management, mild cases need to receive supportive measures, such as stay isolated at home and monitoring up to the end of the isolation measures. Severe cases, however, require clinical stabilization, referral and transportation to referral centers, emergency services or hospital facilities^(^ 8 ^)^.

The increased demand for health services to treat infected individuals has overburdened the health systems in the countries more severely affected by the pandemic. In Brazil, the number of hospitalizations has followed an ascending curve^(^ 9 ^)^, with severe consequences such as overcrowded facilities and exhausted professionals working on the front lines. Hence, many of these professionals, in addition to fighting the COVID-19, are also experiencing a humanitarian crisis, compounded by the scarcity of protective materials, which puts their lives at risk^(^ 10 ^)^.

Health workers providing direct care to COVID-19 patients at the different levels of care are directly and continuously exposed and, therefore, more vulnerable than most of the population. A Chinese study revealed that geographical proximity to the outbreak epicenter directly influenced the severity of COVID-19 cases among these professionals^(^ 11 ^)^.

From the onset of symptoms and signs until the confirmation of the diagnosis, individuals, even health workers, have to follow a path, a journey in which they seek treatment. This journey, which is associated with individual and socio-cultural practices and is intended to solve healthcare problems, is called the therapeutic itinerary^(^ 12 ^)^.

Studies addressing therapeutic itineraries support the understanding of how health services work and behave and how they are used, i.e. the path chosen and its multiple repercussions. A late diagnosis may result when patients are not properly referred^(^ 12 ^)^. This is of concern when such a situation occurs with health workers, that is, people who provide care to other people within the context of health care.

Additionally, because COVID-19 is a disease caused by a highly transmissible virus, having an infected health professional working in a healthcare unit, without having a confirmed diagnosis, may entail consequences, especially because it increases the exposure of other workers and the population seeking health services^(^ 13 ^)^.

Considering these aspects, studying the therapeutic itinerary of health workers diagnosed with COVID-19 is vital. The identification of this itinerary and the difficulties found may help to devise strategies to correct gaps and problems in this itinerary. Hence, this study’s objective was to analyze the therapeutic itinerary of health workers diagnosed with COVID-19.

## Method

This descriptive and exploratory study with a qualitative approach was conducted using an electronic form with health workers diagnosed with the COVID-19, living in the state of Rio de Janeiro, Brazil and working at different levels of care, in public or private healthcare facilities, these being the study inclusion criteria. Individuals with professional training in the health field but not active during the pandemic were excluded.

Data were collected between May 12^th^ and 30^th^, 2020. The workers were invited to participate in the study through social media such as Facebook, Instagram and WhatsApp. The survey was disseminated in the feed and stories of the authors’ Facebook and Instagram accounts, as well as in the accounts of members of research groups and their workgroups in the WhatsApp. These strategies were used to disseminate the study as much as possible and reach a larger number of participants. After accepting the invitation, the individuals clicked on the link provided to receive further information regarding the study and were granted access to a free and informed consent form and the semi-structured questionnaire. Criteria to cease the interviews were theoretical saturation of data^(^ 14 ^)^ and utilization rate index^(^ 15 ^)^.

The semi-structured form was developed by the authors and face and content validated by experts in the field. It contained close-ended questions to characterize the participants including sex, age, profession and preexistent diseases and three open-ended questions: how was the path taken to obtain the diagnosis of COVID-19? What were the aspects that facilitated this path? What were the aspects that hindered it? The participants took approximately 10 minutes to complete the form.

The responses obtained composed the textual *corpus*, which was submitted to lexicographic analysis in *Interface de R pour Analyses Multidimensionnelles de Textes et de Questionnaires* (IRAMuTeQ)^(^ 15 ^)^, via Descending Hierarchical Classification.

The active forms of each class of excerpts were used in the interpretation of data, including nouns, adjectives and unrecognized forms, such as acronyms, with emphasis on the forms that scored ≥3.84 in the Chi-square test (χ^2^), which indicates the associative strength between the words in their respective classes.

The Institutional Review Board approved the study (CAAE 31201420.6.0000.5243 and opinion report 4.012.631). The participants were ensured that their identities would remain confidential. A free and informed consent form was available online. The participants confirmed their consent by checking the option “I read and agree to participate in this survey”. An alphanumerical code was used according to the order in which the participant entered the survey, followed by a code that identified the participant’s profession.

The letter P was used, followed by the number that indicated the participant’s entry order. The acronyms used for the professions were: AG (*agente de saúde* in Portuguese) health agent; AE (*auxiliar de enfermagem* in Portuguese) nursing aid; B (*biólogo* in Portuguese) biologist; D (*dentista* in Portuguese) dentist; EF (*educador físico* in Portuguese) physical educator; E (*enfermeiro* in Portuguese) nurse; F (*farmacêutico* in Portuguese) pharmacist; Fi (*fisioterapeuta* in Portuguese) physical therapist; Fo (*fonoaudiólogo* in Portuguese) speech therapist; M (*médico* in Portuguese) physician; N (*nutricionista* in Portuguese) nutritionist; TE (*técnico de enfermagem* in Portuguese) nursing technician; TL (*técnico de laboratório* in Portuguese) laboratory technician and TH (*técnico de hemoterapia* in Portuguese) hemotherapy technician.

## Results

In total, 132 health workers from 14 different professions participated in this study: health agents (4), nursing aids (1), biologists (2), dentists (2), physical educator (1), nurses (67), pharmacists (2), physical therapists (2), speech therapists (1), physicians (19), nutritionists (1), nursing technicians (28), laboratory technicians (1) and hemotherapy technicians (1).

There were 116 women and 16 men, aged 37.4 years old on average; the youngest was 18 years old and the oldest was 63 years old. Regarding preexisting diseases, 101 denied them and 31 reported at least one, namely: systemic blood hypertension (11), asthma/bronchitis (7), heart disease (3), diabetes (2), rhinitis (2), hyperthyroidism (2), obesity (1), multiple sclerosis (1), depression (1) and hypothyroidism (1).

After processing in IRAMuTeQ, according to basic statistics, the textual *corpus* consisted of 132 excerpts and 5091 words, 741 of which were distinct and 356 were hapax, that is, appeared only once, with an average of 38.5 words *pe*r excerpt.

Segmentation of the *textual* corpus by classes of excerpts and their terms, using the descending hierarchical classification, revealed the central ideas that originated from the participants’ responses. This analysis retained 186 excerpts, classifying 142 of them, with a utilization rate of 76.34%.

**Figure 1:**
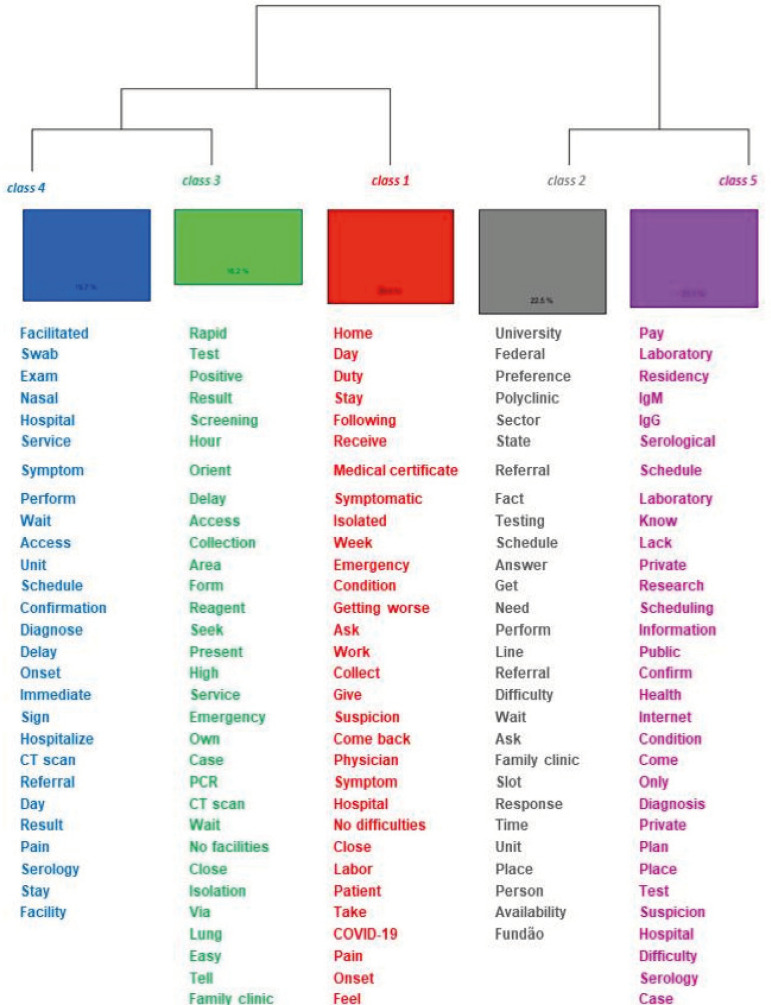
Dendrogram of the descending hierarchical classification of the therapeutic itinerary of health workers diagnosed with COVID-19. Rio de Janeiro, RJ, Brazil, 2020

The grouping of excerpts with similar and associated vocabulary led to the establishment of five stable classes, illustrated on the dendrogram (Figure 1), which presents the relationships between the classes and percentage of each regarding the total *corpus* analyzed.

In the dendrogram, the textual *corpus* was divided into two *subcorpus*. The first was composed of Class 1, which appears in red, (20.4%) and a second subgroup with Classes 4 in blue (19.7%) and 3 in green (16.2%). The second *subcorpus* was composed of Classes 2 in gray (22.5%) and 5 in lilac (21.1%). The classes were named according to their semantic content.

*Class 1 - The beginning of the therapeutic itinerary based on the symptoms.* This class retained 29 excerpts, which correspond to 20.4%. The active forms with Chi^2^≥3.84 in decreasing order are home, day, on_duty, stay, next, receive, medical certificate, symptomatic, isolated, week, emergency, condition, getting_worse, ask, work, collect, give, suspicion, come_back, physician, symptom, hospital, no_difficulties, close, labor, patient and take.

The health workers emphasized that, after the onset of symptoms such as headache, fever, diarrhea, dyspnea, and pain, they sought care in the facilities where they worked, in emergency rooms or primary health care units; furthermore they were isolated themselves at home when suspected of the disease:

*I went to the emergency room of the hospital where I work and after the physician verified the possibility of COVID-19, she gave me a medical certificate and asked me to seek the occupational health sector* (P8, E). *I went to an emergency room due to malaise and headache. They first diagnosed me with sinusitis. When I had fever and diarrhea I went to another service and was isolated under the suspicion of COVID-19* (P47, E). *After my shift, I started having a sore throat, which seemed like an allergy, but because I’m from the health field and work with cancer patients, I decided to stay away* (P85, M). *I presented the first symptoms when I was on duty, I was medicated and isolated myself at home on the following day. Three days after I went to a primary health care unit and they prescribed mandatory isolation* (P108, TE).

The need to confirm the diagnosis and take a sick leave from work led workers to go to different services. Additionally, some highlighted that they were unable to test their families while others obtained a confirmation of the diagnosis after their condition worsened:

*Four days after the onset of symptoms, I went to a federal hospital where I’m a resident and went through the screening process. They scheduled my test for the following day and gave me 7 days off. I still had symptoms after 7 days and the result of the test had not been released yet. So, I needed to go to a private emergency room to get a new medical certificate considering I could not work like that* (P114, E). *Because I’m a hospital employee, they collected my PCR* [*Polimerase Chain Reaction*] *on the fourth day. It happened in a private hospital but I was unable to test my family members who live with me and had symptoms* (P24, M). *The following day, I went to a private hospital and they classified my case as sepsis due to the more intense symptoms, so I was transferred to the ICU* [Intensive Care Unit] *of another hospital* (P59, TE).

*Class 2 - Referral to access the test and confirm COVID-19.* This class retained 32 excerpts, which corresponds to 22.54%. The active forms with Chi^2^≥3.84 in decreasing order include university, federal, referral, polyclinic, sector, state, refer, fact, testing, schedule, achieve, perform, need, line, referral, difficulty.

The participants reported they were referred to testing sites, such as a federal university hospital and state polyclinic linked to the university that is a referral site for the testing of health workers, which, however, did not prevent difficulties such as long waiting time, the need to schedule the test, queues, and distance:

*The federal hospital where I work scheduled the PCR in the federal university. The manager scheduled the test; there was a large number of people waiting for it* (P22, M). *I took the test in the polyclinic, didn’t need to schedule but I had to arrive very early to get it. I got the result by e-mail almost 30 days later* (P37, E). *I got tested in the referral polyclinic because I’m a physician at the university and waited 7 days for the result* (P87, M). *Long waiting both to take the exam and to get the results* (P61, TE). *The hospital where I work does not have the test, so I had to go to a unit far away from my home and had to leave home at daybreak to be able to take the test because there were very few slots* (P65, E).

*Class 3 - Difficulty to access the test and confirm COVID-19.* This class retained 23 excerpts, which corresponds to 16.2%. The active forms with Chi^2^ ≥3.84 in decreasing order include rapid, test, result, hour, screening, orient, delay, access, collection, area, reagent, form and seek.

In this class, the workers talked about the difficulty to access the tests, including the need to go to different places, as well as the long waiting time to collect the material and get the result:

*I had to try to access the test in five different places; I couldn’t stand it so I went to the emergency room* (P5, TE). *I waited five hours to take the test, went through screenings, lectures, data collection and the test. Then I waited another four days to get the result* (P65, E). The delay in getting the results is a huge difficulty (P48, TE). *I waited two hours for assistance and swab collection, with a positive result that was released on the following day. On the same day, I took the rapid test and it was negative* (P8, E).

Given these difficulties, two workers highlighted that they managed to take the test only because of their close personal contacts:

*An acquaintance facilitated my access to the test because the hospital was not testing for non-severe cases* (P8, EF).*I took the test in a health unit where I’m a medical resident; the physician in the staff who organized the schedule helped me out* (P86, M).

*Class 4 - Aspects that facilitate access to the exams in hospital facilities.* This class retained 28 excerpts, which correspond to 19.7%. The active forms with Chi^2^≥3.84 in decreasing order are facilities, swab, leave, exam, nasal, hospital, service, symptom, perform, wait, access, unit, communication and schedule.

The participants mentioned that aspects that facilitated the process include being able to take the tests in the facility where they work, based on their symptoms, without having to wait for them:

*The hospital where I work is testing. So I tested there and the result came in three days* (P30, E). *I went to a private hospital where I work and they collected the swab for COVID-19; I was able to test in the place where I work* (P57, M). *I sought care in the place where I work to take the swab test. I had no difficulties because I work in hospitals that offer tests for employees with a flu condition* (P76, E).

The participants also reported the hospitals’ problem-solving capacity as a possibility to perform other exams such as the CT scan (Computed Tomography Scan):

*Arterial blood gas analysis, d-dimer, chest CT scan, and swab-based collection were performed in the emergency room. I immediately got the result concerning the chest CT scan, but the oropharynx swab took a long time* (P21, M). *I took a CT scan that showed viral pneumonia, the swab took 5 days for the results to be released* (P45, E)*. According to the chest CT scan and swab* (P102, D).

*Class 5 - Difficulties related to misinformation and the cost of tests to confirm the COVID-19.* This class retained 30 excerpts, which correspond to 21.1%. The active forms with Chi^2^≥3.84 in decreasing order are pay, laboratory, home, IgM, IgG (Ig=immunoglobulin), serological, schedule, laboratory, know, lack, private, research, scheduling, information, public, confirm, and health.

In this class, the workers reported a lack of information regarding the test sites available to obtain diagnostic confirmation of COVID-19:

*I don’t know what the correct order is. The exams should have been performed when the first symptom appeared* (P2, E). *I searched on the Internet and made some telephone calls; there is a lack of information* (P6, TE).

Another important difficulty was the poor availability of these exams in the public system. As a result, the participants had to pay for them in private laboratories, even though some of these professionals had few resources or wait for a longer time to have the disease confirmed:

*I took the swab-based collection in a private laboratory because I couldn’t take it in a public hospital* (P12, E). *I hired a private laboratory that collected the swab at my home because the polyclinic took a long time to schedule it, so I ended up hiring a private laboratory. The polyclinic scheduled the exam only after I already had received the test result* (P12, E). *I went to the public system and couldn’t do it; I had to pay even though I couldn’t afford it. There is no respect. If we are not feeling well, we need to know whether we contracted the virus* (P49, TE). *I had to wait 30 days after the onset of symptoms to take the test because there was a lack of tests in the region where I live* (P89, D).

Coupled with these difficulties is the fact that health insurance/plans do not pay for the tests at an outpatient level, even for health workers. Additionally, even when the workers pay for the expenses, getting a slot is not always certain, so there is a need to seek different services:

*The hospitals only perform the tests among hospitalized patients and the health insurance/plans do not cover for outpatient tests. You have to pay and wait to schedule a test because the immediate test is only available for physicians* (P45, E). *I tried to schedule it in different places with no success; there were no slots in the private laboratory* (P41, E).

A lack of slots in public services to take the test led workers to seek alternatives to overcome difficulties:

*I took the serological test to search for IgG and IgM antibodies because a supplier of laboratory diagnostic products did the courtesy* (P80, B). *The director of the place where I work surveyed the employees. I took the serology and the swab tests, otherwise, I believe I wouldn’t have known that I was contaminated and could have infected other people* (P99, TE).

## Discussion

The results show that the therapeutic itinerary of the workers participating in this study to access the COVID-19 test is marked by having to go to different health facilities, not only to confirm the disease but also to obtain a medical certificate, which extends the time between the suspicion and confirmation of the condition.

Due to the risk of infection and the possibility to spread COVID-19 to other people, including family members, ensuring that care is provided to health workers and rapidly performing the tests among individuals with symptoms are essential^(^ 16 ^)^. Therefore, recommendations that should not be neglected during the pandemic include giving priority to health professionals working on the front line^(^ 17 ^-^ 18 ^)^.

The results, however, reveal a lack of protocols or failure to follow institutional protocols to guide and ensure health workers with a suspicion of being infected with the disease receive proper care, as well as to manage exposure to the virus, actively monitoring respiratory signs and symptoms and notifying health governmental and the institutions’ health occupational authorities.

The WHO provides guidelines, among which taking sick leave and ensuring that all workers with suspicion of being infected with COVID-19 are tested stand out. The institution should be responsible for managing the workers, whether they test positive or not^(^ 19 ^)^, a procedure not verified, absolutely, among all those reporting their therapeutic itinerary.

In Brazil, health workers with suspicion of flu syndrome, that is, fever accompanied by a cough or sore throat or respiratory problems, are supposed to take immediate leave and resuming work only under certain conditions. Hence, when RT-PCR (Reverse Transcriptase - Polimerase Chain Reaction) or serological tests are available, returning to work is allowed if the test is negative, considering the specificities of each of them. When there are no tests available, the worker is supposed to remain on leave for at least 72 hours if asymptomatic and at least 7 days after the onset of symptoms. A surgical mask must be used for, at least, 14 days after the onset of symptoms. If testing positive, recommendations include staying isolated at home for 14 days after the beginning of the symptoms^(^ 8 ^)^.

When referred to testing sites, the workers had to queue, long waiting times, had difficulties to schedule the test, while in some cases, the services were located far away from their homes. These aspects concerning organizational accessibility were also reported in a study addressing the patients’ satisfaction with a primary health care service^(^ 20 ^)^.

Considering the different difficulties perceived in the itinerary of this study’s participants, working in facilities that provide tests to their employees promotes a faster diagnosis of COVID-19, resulting in greater safety for them, their families and patients. Additionally, it helps to control the disease better and is one of the main elements that facilitate the therapeutic itinerary.

Therefore, in the search for diagnostic confirmation, some workers highlighted they sought a hospital given its problem-solving capacity to perform exams and release results fast, such as CT scans. This exam gives the possibility to perform a global assessment of infected patients. Even though little specific, it is sensitive to show the diseases’ most frequent findings in the lung^(^ 21 ^)^, such as bilateral lung lesions, ground-glass opacity and air bronchogram^(^ 22 ^)^. This exam, however, was restricted to those who had access to hospital facilities, that is, it was not available to all the workers.

In addition to the little availability of tests in the public system, which was reported as an aspect that hindered the therapeutic itinerary, the participants reported there was a lack of information regarding the tests. The RT-PCR is a virological test (genetic material or antigens). It detects the presence of viral components, confirming the diagnosis of people with symptoms that are compatible with COVID-19. It is indicated for populations at high risk of infection, such as symptomatic health workers and those who have morbidities/severe conditions, among which hypertension, diabetes, obesity, cardiovascular problems, respiratory history or immunosuppression, etc. It is also used to verify whether an individual has recovered from COVID-19^(^ 23 ^)^.

Diagnostic confirmation is based on the molecular detection of the viral genome (RNA -ribonucleic acid- detection) or its proteins (antigens). Thus far, it has been possible to detect the virus, at least, 48 hours before the onset of symptoms (pre-symptomatic) up to 12-14 days. Collection in the upper respiratory tract is recommended between 6-7 days (nasal swab/oropharyngeal) and up to 20 days (or more) in the lower respiratory tract, including sputum, tracheal aspirate and bronchoalveolar washing^(^ 23 ^)^. Even though the RT-PCR remains detectable in some individuals from 2 to 6 weeks, most cases represent inactive genetic material, not presenting the risk of transmission^(^ 16 ^)^.

The serological tests detect levels of IgM, IgA and IgG antibodies as part of the individuals’ immune response against the SARS-CoV-2 virus, that is, indicate a previous or ongoing contact^(^ 23 ^)^. These tests are performed using different techniques, such as automated chemiluminescence immunoassay, immunoenzymatic assay, and immunochromatography, that is, a rapid, less sensitive test. In general, these tests present between 60% and 70% sensitivity around the 7^th^ day and around 90%, 10 days after the onset of symptoms. Hence, a serological negative result during the first seven days of the disease cannot be used as a criterion to rule out a case^(^ 16 ^)^.

Still, regarding the serological test, the presence of IgM or IgA indicates acute infection while the presence of IgG indicates prior contact with the SARS-CoV-2, which may be related to having immunity against the virus. Whether antibodies confer immunity against the virus is under investigation. Health workers with positive IgG should wear personal protective equipment (PPE) when in contact with suspected or confirmed cases^(^ 16 ^)^.

In this Brazilian context, given the insufficient number of molecular tests to meet the demand, a fact that is reinforced by this study’s participants, many workers had to pay for the tests performed in private laboratories. The Brazilian Ministry of Health (MH) established criteria for performing rapid serological tests, determining that health workers and public security workers had priority because these are the most frequently exposed to the coronavirus in addition to their contacts at home. Hence, the MH asked that the states and cities tested symptomatic active workers as well as those living in the same household with the flu syndrome. A blood sample is used for the rapid test and the result is released in approximately 20 minutes^(^ 8 ^)^. As previously mentioned, the problem of this type of test is that greater sensitivity is obtained only 10 days after the onset of symptoms^(^ 16 ^)^.

Despite the MH’s recommendations, workers still faced difficulties to take the test in the public system due to various reasons, such as lack of inputs or because they did not present a severe condition. Additionally, some participants reported doubts and divergences between the results obtained with the RT-PCR and rapid tests, a fact that may be explained by the need to perform the tests within the period in which sensibility is greater. Additionally, even though the rapid tests to detect antibodies are widely produced and sold, the quality of these tests varies, as they do not reveal the nature of the antigens used. These are qualitative tests and, only, indicate the presence or absence of SARS-CoV-2 antibodies^(^ 24 ^)^.

The doubts the workers reported regarding the type of test, ideal period to take it, among others, indicate a need to providing training not only to address diagnostic tests but, also, to decrease the risk of infection, considering the vulnerability of these individuals to COVID-19. Therefore, topics such as how to properly wear PPE, hand hygiene, health waste management, sterilization of patient care devices and management of occupational exposure^(^ 25 ^)^ should be addressed.

Because the Primary Health Care (PHC) is the entry door to the Unified Health System (SUS - *Sistema Único de Saúde* in Portuguese), it works on decentralizing care, testing suspicious cases and active search and monitor positive cases. These actions favor epidemiological surveillance and the planning of local control measures^(^ 26 ^)^. The workers did not frequently report PHC units as testing sites though. Instead, hospitals and polyclinics, especially those linked to universities, were the facilities most frequently reported.

Hence, PHC units should take an active role in terms of taking measures such as reorganizing the flow of patients in the services and improving the units’ physical structures. Additionally, the MH needs to give priority to PHC in its agenda, considering that the future of SUS and the health of Brazilians depend on it^(^ 27 ^)^.

This study’s limitations include the fact it was conducted in a single Brazilian state. Considering that the number of cases advanced in other states, new studies are needed to fill in gaps concerning the therapeutic itinerary of health workers from other Brazilian regions.

As for the advancement in knowledge, this study reveals that the difficulties experienced by health workers during their therapeutic itinerary were mostly associated with a lack of inputs in the public system to deal with the beginning of the pandemic in the Brazilian context, especially in terms of availability of tests, initially designated only for the most severe symptomatic cases. Hence, to more rapidly access the tests or care, those working in referral facilities took part in the institution’s survey, received help from personal contacts, or paid for the tests in the private system.

Given the virus’s transmissibility and the vulnerability of health professionals working on the front line, these workers should have priority in the access to tests and enjoy faster processes in terms of testing, assessment, monitoring and sick leave whenever there is a suspicion or confirmation of infection by SARS-CoV-2. The reason is that, besides ensuring that care is provided, these strategies can minimize negative impacts, such as having a massive number of workers on the front line being required to stay home, as well as spreading the virus within care settings, or to family contacts.

## Conclusion

Regarding the therapeutic itinerary of the health workers diagnosed with COVID-19, the results show the difficulties health workers faced to access tests, obtain information regarding tests, to access the results and to obtain a medical certificate to stay isolated at home. The study shows an active search on the part of professionals who worked in a facility that did not manage or monitor such cases.

Hence, this study revealed a therapeutic itinerary marked by difficulties that included delayed diagnoses, lack of precise information, uncertainties and having to pay for the tests. These results indicate the urgent need to reorganize the services and institutions to manage and monitor cases of occupational exposure. Despite restricted resources in institutional contexts and the growing demand for diagnostic tests and monitoring of the entire population, there is a need to protect and improve the therapeutic itinerary of health workers, considering that they are essential to fight the pandemic.
